# Is Acupuncture Effective for Hypertension? A Systematic Review and Meta-Analysis

**DOI:** 10.1371/journal.pone.0127019

**Published:** 2015-07-24

**Authors:** Xiao-Feng Zhao, Han-Tong Hu, Jia-Shen Li, Hong-Cai Shang, Hai-Zhen Zheng, Jian-Fei Niu, Xue-Ming Shi, Shu Wang

**Affiliations:** 1 Institute of Acupuncture and Moxibustion, First Teaching Hospital of Tianjin University of Traditional Chinese Medicine, Tianjin, China; 2 Tianjin University of Traditional Chinese Medicine, Tianjin, China; 3 Department of Acupuncture and Moxibustion, First Teaching Hospital of Tianjin University of Traditional Chinese Medicine, Tianjin, China; 4 Key Laboratory of Acupuncture of Tianjin, First Teaching Hospital of Tianjin University of Traditional Chinese Medicine, Tianjin, China; National Taiwan University, TAIWAN

## Abstract

**Objective:**

To determine the efficacy of acupuncture for hypertension.

**Method:**

Seven electronic databases were searched on April 13, 2014 to include eligible randomized controlled trials (RCTs). Data were extracted and risk of bias was assessed. Subgroup analyses and meta- analysis were performed.

**Results:**

23 RCTs involving 1788 patients were included. Most trials had an unclear risk of bias regarding allocation concealment, blinding, incomplete outcome data and selective reporting. Compared with sham acupuncture plus medication, a meta-analysis of 2 trials revealed that acupuncture as an adjunct to medication was more effective on systolic (SBP) and diastolic (DBP) blood pressure change magnitude (n=170, SBP: mean difference (MD)= -7.47,95% confidence intervals (CI):-10.43 to -4.51,I^2^ =0%; DBP: -4.22,-6.26 to -2.18, 0%).A subgroup analysis of 4 trials also showed acupuncture combined with medication was superior to medication on efficacy rate (n=230, odds ratio (OR)=4.19, 95%CI: 1.65 to 10.67, I^2^ =0%). By contrast, compared with medication, acupuncture alone showed no significant effect on SBP /DBP after intervention and efficacy rate in the subgroup analysis. (7 trials with 510 patients, SBP: MD=-0.56, 95%CI:-3.02 to 1.89,I^2^ =60%; DBP: -1.01,-2.26 to 0.24, 23%; efficacy rate: 10 trials with 963 patients, OR=1.14, 95% CI: 0.70 to 1.85, I^2^ =54%).Adverse events were inadequately reported in most RCTs.

**Conclusion:**

Our review provided evidence of acupuncture as an adjunctive therapy to medication for treating hypertension, while the evidence for acupuncture alone lowing BP is insufficient. The safety of acupuncture is uncertain due to the inadequate reporting of it. However, the current evidence might not be sufficiently robust against methodological flaws and significant heterogeneity of the included RCTs. Larger high-quality trials are required.

## Introduction

Essential hypertension (EH), which affects up to one billion individuals worldwide and is attributable each year for more than 7 million deaths and loss of 64 million disability-adjusted life years (DALYs) [1], remains a major public health challenge in both developed and developing countries despite all the progress in prevention and management of it [2].

Antihypertensive medication serves as a major therapy for treating hypertension. However, due to various side effects or safety concerns, such as drug resistance which could affect therapeutic efficacy, this therapy is far from satisfactory. Thus, seeking for an effective and less harmful treatment becomes an important goal for treating EH.

As a non-pharmacological intervention, acupuncture is an ancient Chinese therapy by inserting needles to the acu-points on the body surface along meridians to treat a wide range of diseases. Case reports and trials with small sample sizes suggested that acupuncture was an effective therapeutic intervention for treating EH [3]. It could be used on patients who want to avoid drug therapy or as an alternative option to reduce dosages of antihypertensive agents. But two previous systematic reviews claimed that the evidence of acupuncture for lowering blood pressure (BP) was inconclusive, mainly due to the paucity of rigorous trials [4, 5]. However, both of them failed to include all relevant RCTs published in China. And their literature searches were performed up to 2010. Several new RCTs have since been published. And though in the past two years, another 2 systematic reviews have been published. One of them [6] only included 4 randomized sham-controlled clinical trials and the other one [7] included sufficient trials but not all of them were rigorously randomized controlled trials (RCTs).And there were some flaws in this review in term of methodology, data extraction and eligibility of included RCTs as well [8]. And there was another systematic review to evaluate the long-term efficacy of acupuncture on hypertension but it just included Chinese RCTs [9]. Some quasi-RCTs were also included in the review so its conclusion may not be solid. Thus, it is timely to update the evidence whether acupuncture could treat EH. Therefore, we conducted this systematic review to critically assess all the currently rigorous RCTs to evaluate the efficacy of acupuncture for hypertension. And safety evaluation of acupuncture was assessed as well.

## Methods

The PRISMA checklist is available as supporting information; See S1 Appendix.

### Database and Search strategy

We searched the following databases from their inception through April 13, 2014: PUBMED, EMBASE, Cochrane Central Register of controlled trials, International Clinical Trials Register Platform of WHO, Chinese Scientific Journal Database, China National Knowledge Infrastructure (CNKI), and Chinese Evidence-Based Medicine Database. The following search terms were used individually or jointly: ‘hypertension’, ‘essential hypertension’, ‘blood pressure’, ‘acupuncture’, ‘electroacupuncture’, ‘clinical trial’, and ‘randomized controlled trial’. No language restrictions were imposed. The search strategy for each database is available in S2 Appendix. We also carefully scanned the references of all the eligible articles of RCTs to identify further publications.

### Inclusion Criteria

RCTs which involved acupuncture as a therapeutic intervention for treating hypertension were included. Patients were diagnosed as hypertensive, with a systolic BP (SBP) ≥140 mm Hg and/or a diastolic BP (DBP)≥90 mm Hg. All RCTs should meet the following criteria: (1) sham and/or active control procedure was used (2) primary outcomes included BP before and after treatment, or BP change magnitude between baseline and post-intervention, or efficacy rate.

Exclusion criteria was as below: (1)pseudo-randomized trials simply claimed to be randomized with totally no description of the method of random sequence generation.(2) RCTs using laser acupuncture, acupressure, and transcutaneous electrical nerve stimulation (without needing insertion) as controls or using interventions whose efficacy is not yet established (i.e., herbal medicine) in the control group. (3) trials which failed to offer proper data to extract.

### Data Extraction

Two reviewers (Hai-Zhen Zheng, Jia-Shen Li) independently evaluated all the eligible articles for inclusion, followed by data extraction in term of author, country, participants, acupuncture treatment, control types, treatment course, sessions of treatment, primary and secondary outcomes(i.e., SBP/DBP after intervention, SBP/DBP change magnitude, efficacy rate and adverse events), using a pre-defined data extraction form (Table 1). Disagreements were resolved by consensus or arbitration by a third reviewer (Xiao-Feng Zhao). All continuous data regarding BP which was presented as mean ±standard deviation (SD) were extracted if reported. Missing data or further information was sought from the primary authors via email if necessary.

**Table 1:** Characteristics and main outcomes of Included Studies.

Author	Hypertension classification	N(male/female); Age(range of age); Intervention/control		Intervention	Acupuncture points	Control	Treatment Course	Sessions of treatment	Duration of one session	Background of acupuncturists	Main Outcome Measurement
Dongjie Zhao(2003)[12]	EHT, stage I and stage II	30(19/11);40.30y(34-57y)	30(18/12);46.10y(40-62y)	Acupuncture plus lifestyle modification	CV4,ST36, ST40,SP6, LR3	Lifestyle modification	40d	30	20min	NR	BP before and after intervention
Qingming Wu(2003)[13]	EHT, stage I and stage II	40(23/17);48.55y	40(21/19)47.85y	Acupuncture	LI4,LR3, GV20	Captopril	30d	30	20min	NR	BP before and after intervention
Jin Chen(2006)[14]	EHT	30(12/18);72y(58-85y)	30(16/14);68.6y (45-84y)	Abdominal acupuncture plus antihypertensive medicine	CV12,CV10,CV6,CV4,ST24,ST26,CV8	Tailored antihypertension	7d	5	30min	NR	1.Efficacy rate 2.BP before and after intervention
Jun Chen(2010)[15]	EHT, stage I	30(14/16);48.2y (39-71y)	30(17/13);50.5y (35-69y)	Acupuncture plus antihypertensive medicine	GB20,LR2	Felodipine	15d	15	30min	NR	Efficacy rate
Chaohui Zhang(2004)[16]	EHT, stage I and stage II	30(22/8);56.50y	30(20/10);55.50y	Acupuncture	GB20,LR2	Compoundserpine	30d	30	30min	NR	Efficacy rate
Dianhui Yang(2010)[17]	EHT, stage I and stage II	30;(28- 44y)	30;(30- 45y)	Electric acupuncture	LR3,LI11	Captopril	14d	14	30min	NR	BP before and after intervention
Yuhong Guo(2007)[18]	EHT, stage I and stage II	40(22/18);43.83y(32-64y)	40(23/17); 44.2y(29-65y)	Acupuncture	GV20,LI11,ST40,LR3,KI3,ST36,SP6	Enalapril	30d	30	30min	NR	BP before and after intervention
Macklin(2006)[19]	EHT with BP after suspension of antihypertensive medications between 140/90 mm Hg and 179/109mmHg	Ind:64 (34/30); 56.8y;Std:64(35/ 29); 55.9y	64(35/29);53.2y	Acupuncture	Individualized acupuncture* Standard acupuncture* and auricular points*	Sham acupuncture	42d	12	30min	YES	1.BP change magnitude 2.Adverse events
Xiaoqing Lee(2008)[20]	EHT, stage I and stage II	67(36/31);59.50y	65(39/26);58.18y	Acupuncture	LR3	Captopril	7d	7	20min	NR	1.Efficacy rate 2.Adverse events
Kim[21]	EHT, stage I	12(8/4);52.08±8.69y	16(8/8);52.38±10.3y	Acupuncture	ST36,PC6	Sham acupuncture	56d	16	20min	YES	1.BP before and after intervention 2.BP change magnitude
Yan Wei(2006)[22]	EHT	40(21/19);57.95y	Group1: 40(23/17);55.36y;Group2:40(22/18);58.14y	Acupuncture	ST9,GV20,LI11,LR3,KI3	Captopril	45d	45	Not retaining needle	NR	Efficacy rate
Wenjun Wan(2009)[23]	EHT, stage I and stage II	30(19/11);63.72y(45-68y)	30(17/13);65.24y(46-67y	Electric acupuncture	LI11	Nicardipine	15d	15	10min	NR	1.BP before and after intervention 2. Efficacy Rate
Tao Qu(2009)[24]	EHT, stage I and stage II	127(69/ 58);55.43y	125(57/ 68);55.24y	Acupuncture	GB20	Betaloc	28d	28	30min	NR	Efficacy rate
Bangguo Chen(2006)[25]	EHT, stage I and stage II	30(20/10);54.75y(36-65y)	30(21/9);51.72y (33-61y)	Acupuncture	GB20	Betaloc	28d	28	30min	NR	1. Efficacy rate 2.BP before and after intervention
Fan Huang(2007)[26]	EHT, stage I and stage II	30(14/16);56.51y(45-70y)	30(13/17);58.12y(47-70y)	Acupuncture plus Captopril	GB20,LI11, PC6,ST36, ST40,LR3	Captopril	28d	28	30min	NR	1.Efficacy rate 2.BP before and after intervention
Guoxiang Feng(2003)[27]	EHT, stage I and II	30(18/12);47.35y	30(14/16);48.35y	Acupuncture	LI4,LR3,GV20	Captopril	30d	30	20min	NR	Efficacy rate
Zhikun Shen(2007)[28]	Resistant hypertension, stage I,II and III	25(15/10);57.32y(45∼76y)	25(16/9);58.21y(44∼79y)	Acupuncture plus Nifedipine	ST 36	Nifedipine	25d	20	30min	NR	1.Efficacy rate 2.BP before and after intervention
Yue Yang(2010)[29]	EHT	20(none);none	generalized acupuncture:20(none);none; medicine:20(none);none	Acupuncture	ST9,LI4, LI11,ST36,LR3	Medicine: hydrochlorothiazide; Generalized acupoints:ST9,LI11, SP6,ST36, GB20	21d	21	NR	NR	Efficacy rate
Chaoyang Ma(2011)[30]	EHT, stage I and II	40(25/15);66.39y	40(22/18);64.58y	Electric acupuncture	LI11	Nicardipine	15d	15	10min	NR	1.BP before and after intervention 2.Efficacy rate
Hui Liao (2006)[31]	EHT, stage I and II and III	59(31/28);56.5±7.9y	31(17/14);55.6±8.6y	Acupuncture	PC7, PC6, LR2, LR3, LR8, KI3, KI7, BL60, LI11, ST40	Captopril	14d	28	30min	NR	1. Efficacy rate 2.BP before and after intervention
Kraft K(1999)[32]	mild hypertension	7	7	Acupuncture	BL18,BL23,GB20,BV20, HT7,KI3, LR2,LR3,SP6	Sham acupuncture	42d	12	3min	NR	BP change magnitude
Flachskampf FA(2007)[33]	mild or moderate arterial hypertension SBP:140-179mmHg DBP:90–109> mmHg	72(39/33);58.80y	68(27/41);58.00y	Acupuncture plus antihypertensive medication	LR2,LR3,LI11,LI4,ST36,ST40,BL18,BL23,GB20,EX-HN5,KI3,SP6,SP9,CV4,CV6, CV12	Sham acupuncture plus antihypertensive medication	42d	22	30min	YES	1.BP before and after intervention 2.BP change magnitude 3.Adverse events
Yin C(2007)[34]	SBP≥120 mmHg or DBP≥80 mmHg. Subjects with SBP>140 mmHg or DBP>90 mmHg were included only when they were already on antihypertensive medication.	15(4/11);52y(49- 56y)	15(5/10);54y(51- 57y)	Acupuncture plus antihypertensive medication	ST36,LI11, BL25,SP3, LU9,BL13, KI2,KI7,CV4,LI1,GB20,GV14	Sham acupuncture plus antihypertensive medication	56d	17	Not retaining needle	NR	1.BP change magnitude 2.Adverse events

Abbreviation: EHT, essential hypertension; NR, not report;

* Individualized acupuncture: BL18, BL20, BL23, BL64, CV4, CV6, CV12, GB20, GB21, GB34, GB43, GV4, GV20, HT7, KI3, LI4, LI11, LR2, LR3, PC6, ST6, ST8, ST36, ST40, ST44, EX-HN5, EX-HN3;

*Standard acupuncture:GB20,LI11,LR03,SP06,ST36;

*auricular points: Kindey, Shenmen, Heart, Step-down ditch.

### Assessment for Risk of Bias

Risk of bias assessment of the included RCTs was independently performed by two reviewers (Hai-Zhen Zheng, Jia-Shen Li) using the Cochrane criteria [10]. Disagreements were resolved by discussion. Risk of bias of the following domains were assessed (1) random sequence generation (2)allocation concealment(3)blinding of participants and personnel (4)blinding of outcome assessment(5) incomplete outcome data (6)selective reporting(7)other bias. Considering the characteristic of acupuncture RCTs, other bias was defined as whether the trial reported the method of acupuncture operation, as well as Deqi sensation, a situation in which patients experience a radiating sensation considered to be indicative of effective needling. Our judgements on these domains were categorized as ‘low’ risk of bias, ‘high’ risk of bias or ‘unclear’ risk of bias based on the Cochrane assessment tool [10]. For examples, as for ‘low risk’ of bias for the domain of random sequence generation, the investigators should describe a random component in the sequence generation process such as referring to a random number table; using a computer random number generator or coin tossing.

### Statistical Analysis

Meta-analysis was performed using Review Manager V5.1. Continuous data were presented as mean difference (MD) and its 95% confidence intervals (CI). Heterogeneity was examined using the I^2^ test, where I^2^ values of 50% or more were considered to be indicative of a substantial level of heterogeneity [11]. Fixed effects model was used if there was no significant heterogeneity; random effects model was employed when there was significant heterogeneity. Based on different outcome measures, if significant heterogeneity between studies was detected, we would investigate possible causes from clinical perspectives by conducting subgroup analysis. Various subgroup analyses were performed based on types of interventions (i.e., acupuncture VS western medicine, acupuncture plus western medicine VS western medicine), sample size (i.e., n≤60 VS n>60), sessions of treatment(i.e., i.e., n≤20 VS n>20) and type of stimulation (manual VS electric). Regarding dichotomous data (i.e., efficacy rate), results were presented as ORs with 95% CIs, using either a fixed or random-effects model depending on the statistical heterogeneity. Regarding some studies compared the effect of more than two intervention groups, the overall effects of acupuncture (i.e.,(group1 +group2) VS control) were tested.

## Results

### 3.1 Literature Search

**Fig 1:**
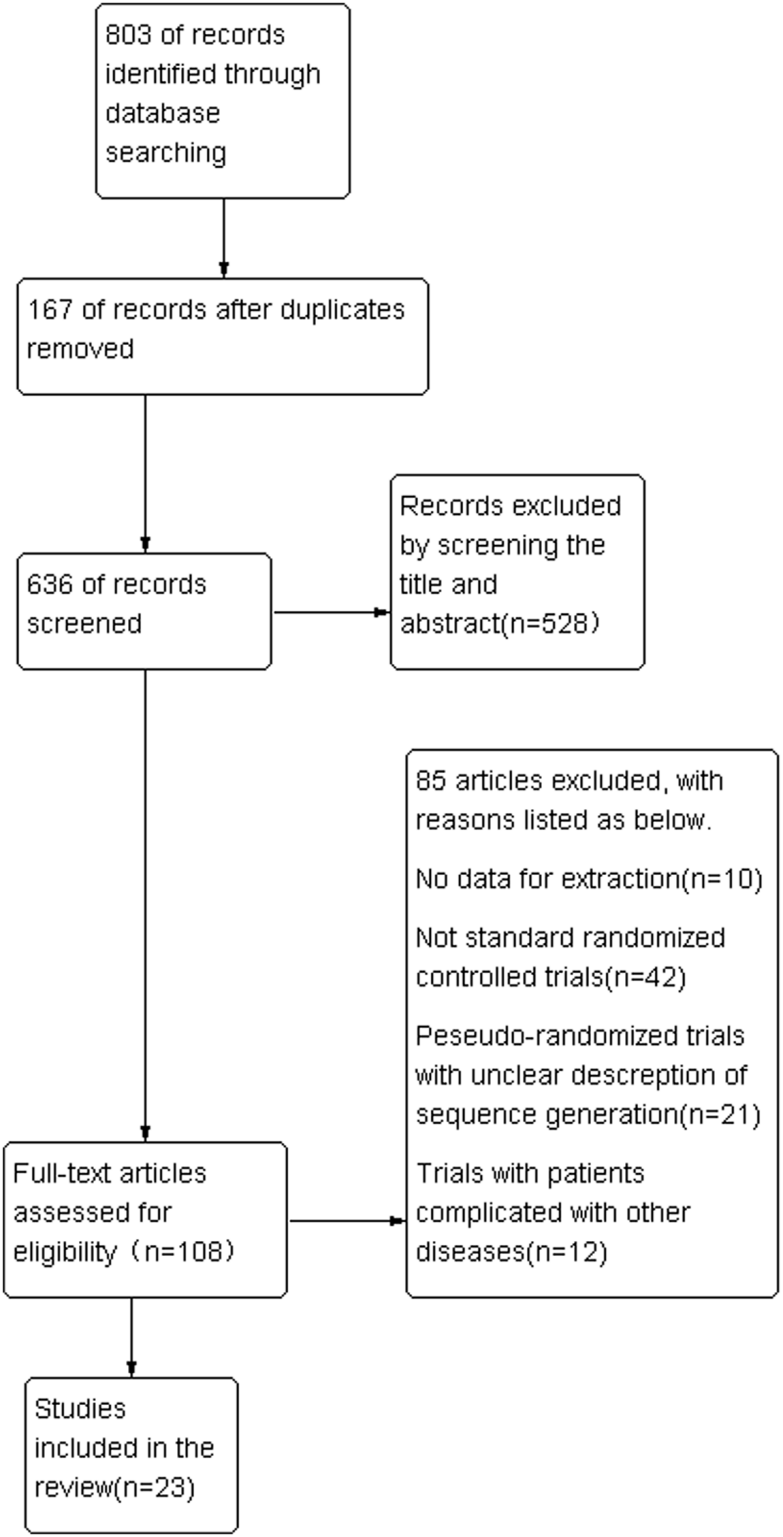
Flowchart of the trial selection process.

The process of literature search was displayed in the flowchart (as shown in Fig 1). After primary search in the target databases, 803 articles were screened. And 528 records were excluded by reading titles and abstracts. Full texts of 108 potentially relevant articles were assessed for eligibility and 85 RCTS of them were excluded by using our inclusion and exclusion criteria. Finally, 23 RCTs were eligible [12–34].

### 3.2 Study Characteristics

The characteristics and main outcomes of each trial were summarized in Table 1. The 23 RCTs included a total of 1788 patients with hypertension, with the average number of 78 per trial, ranging from 14 to 252. A majority of RCTs involved patients with mild or mild to moderate hypertension, except two studies enrolled participants with hypertension of Stage III[28, 31]. And 18 RCTs were conducted in China [12–18, 20, 22–31], 2 in Germany [32, 33], 2 in South Korea [21, 34] and 1 in the United States [19].

Varied acupuncture techniques were used in terms of selection of acupuncture-points, manipulation or stimulation methods. The most frequently used acupuncture points were LR3 (taichong, in 12 trials), LI11 (quchi, 11 trials), GB20 (fengchi, 10 trials), ST36 (zusanli, 8 trials), followed by LI4 (hegu, 5 trials), SP6 (sanyinjiao, 5 trials), CV4 (guanyuan, 5 trials), PC6 (neiguan, 4 trials).Participants received acupuncture treatment 3 to 30 min per session for mean 28.5 days, ranging from 7 to 56 days. 16 trials had a clear description of ‘Deqi’, which was associated with better effectiveness of acupuncture. Only 3 of the 24 trials introduced the background of acupuncturist [19, 21, 33] and the majority of the studied described the manipulation method of acupuncture. Besides, 11 trials reported the method of BP measurement by using 24-hour ambulatory BP monitoring or mercury sphygmomanometer or automated sphygmomanometer.

### 3.3 Types of Interventions

Acupuncture group included acupuncture or electro-acupuncture alone, or combined with western medicine. And one trial involved acupuncture combined with lifestyle modification [12]. 16 trials employed acupuncture as sole treatment, whereas in 6 trials[14,15,26,28,33,34], acupuncture was used as an adjunctive therapy for medication. Types of control groups consist of western medicine, sham acupuncture, lifestyle modification and western medicine combined with sham acupuncture. Sham acupuncture alone was adopted in 3 RCTs [19, 21, 32] and 2 trials [33,34] used sham acupuncture plus western medicine. One study [12] employed lifestyle modification alone as control.

### 3.4 Investigation of Heterogeneity and Subgroup Analysis

**Fig 2:**
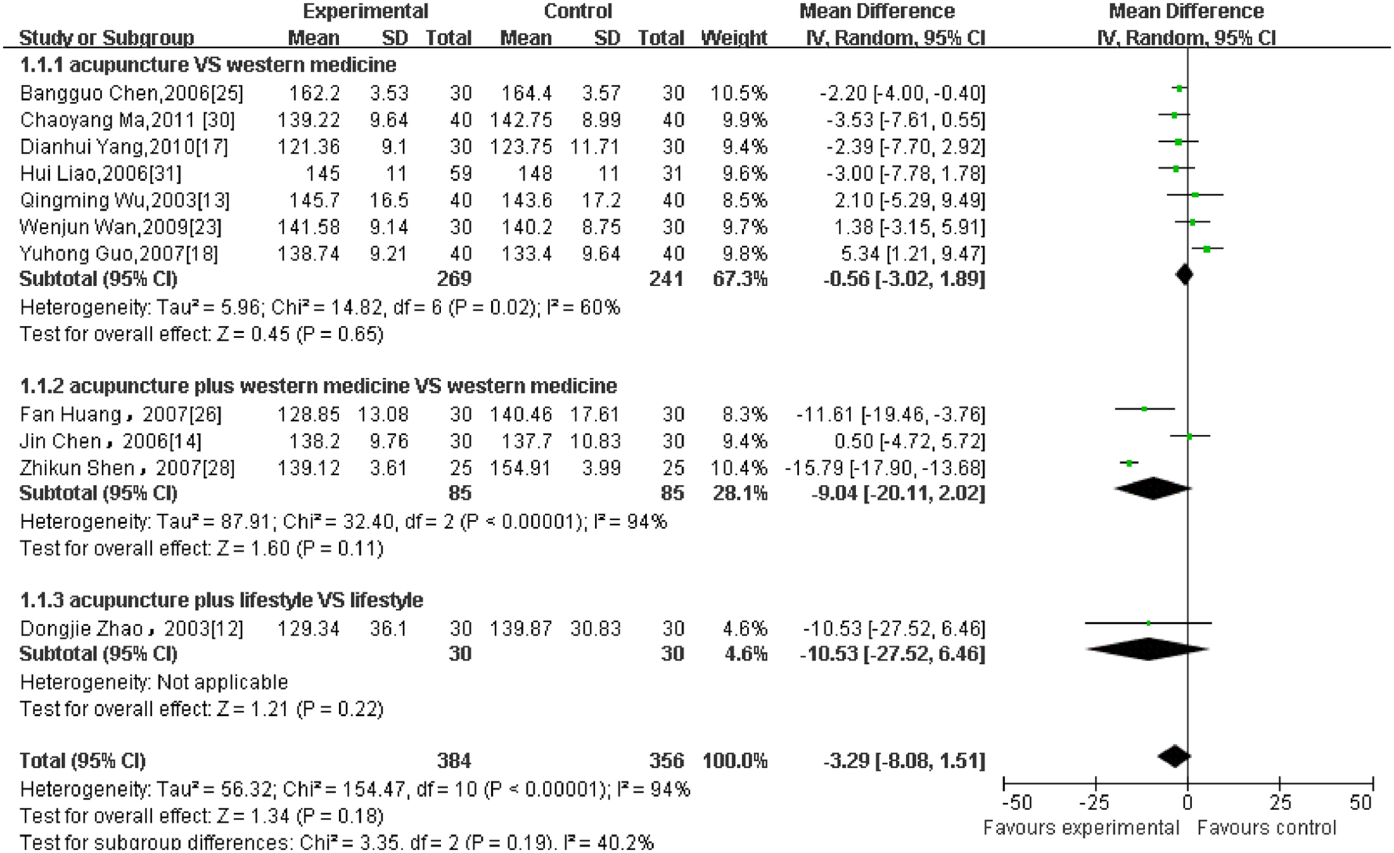
The forest plot of outcome measure ‘SBP after intervention.’

**Fig 3:**
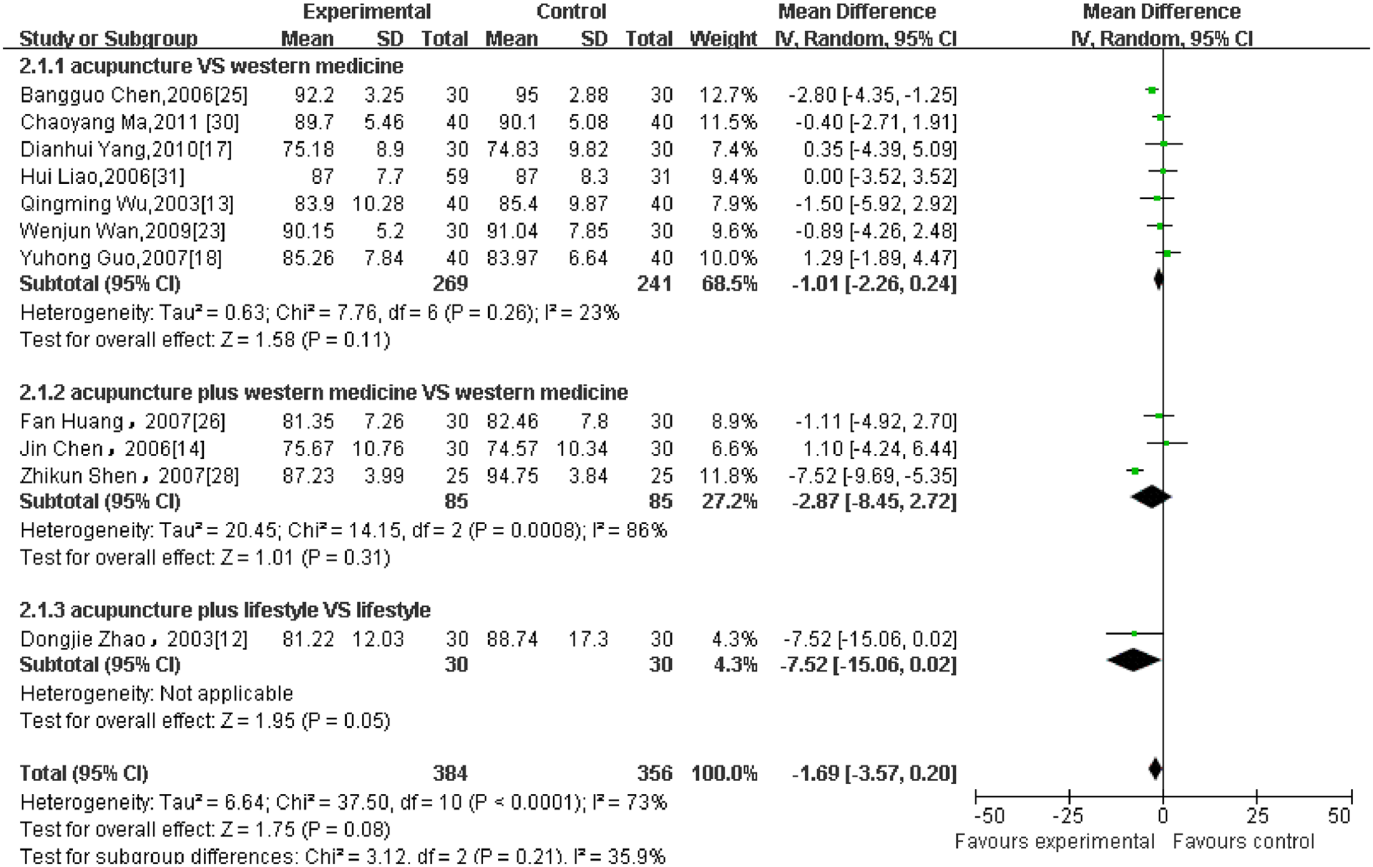
The forest plot of outcome measure ‘DBP after intervention.’

**Fig 4:**
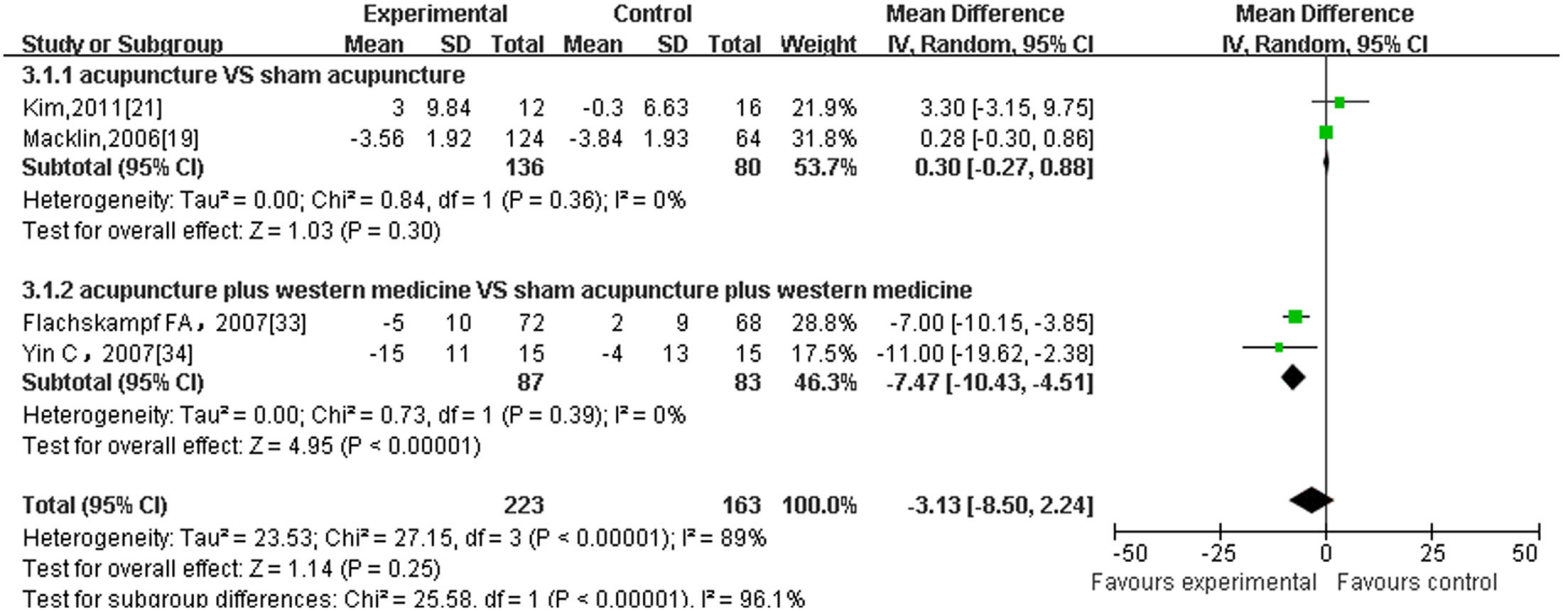
The forest plot of outcome measure ‘SBP change magnitude.’

**Fig 5:**
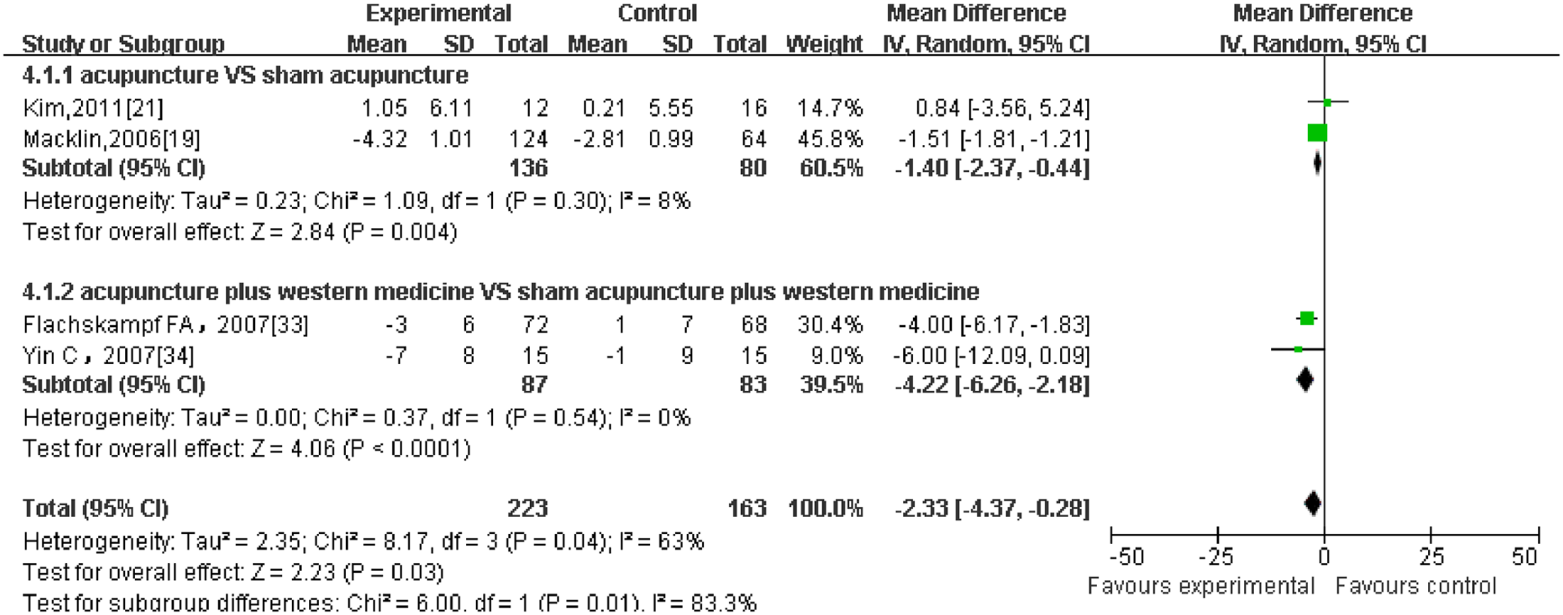
The forest plot of outcome measure ‘DBP change magnitude.’

**Fig 6:**
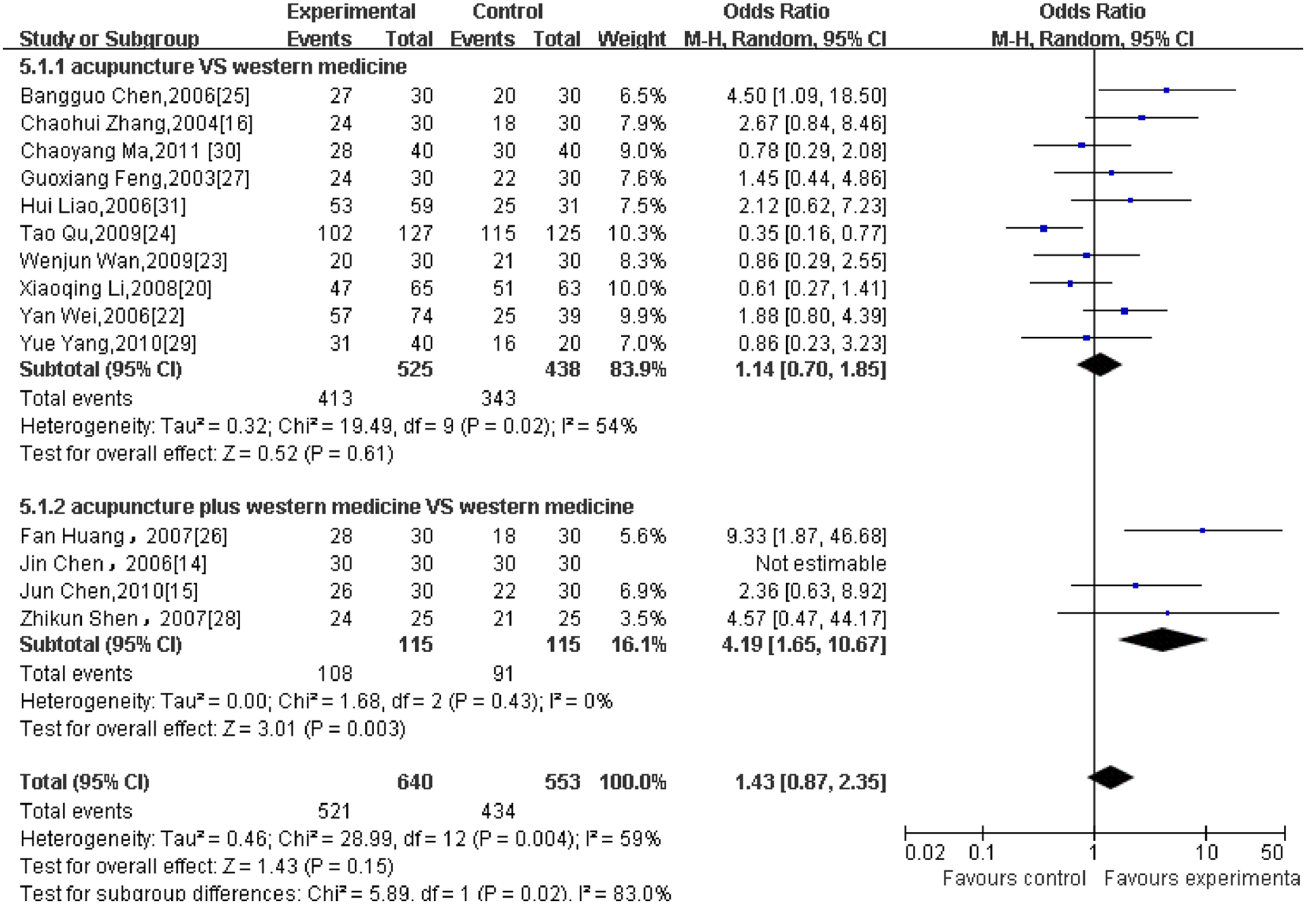
The forest plot of outcome measure ‘Efficacy Rate.’

Regarding different outcome measurements, significant heterogeneity between included RCTs was found (Figs 2–6: I^2^ = 94% for ‘SBP after intervention’, I^2^ = 73% for ‘DBP after intervention’, I^2^ = 89% for ‘SBP change magnitude’, I^2^ = 63% for ‘DBP change magnitude’ and I^2^ = 59% for ‘efficacy rate’, respectively). Possible causes for heterogeneity were searched by performing subgroup analysis. Based on the clinical perspective, a subgroup analysis was performed based on types of intervention (i.e., acupuncture VS western medicine, acupuncture plus western medicine VS western medicine) because this might be one of the main factors causing heterogeneity clinically. Additionally, various subgroup analyses were also performed based on sample size (i.e., n≤60 VS n>60), sessions of treatment(i.e., i.e., n≤20 VS n>20) and type of stimulation (manual VS electric). Results of subgroup analyses were displayed in Tables 2–4. As for outcome measures ‘SBP/DBP change magnitude’, it turned out that types of intervention could be one possible cause for clinical heterogeneity. However, regarding outcome measures ‘SBP/DBP after intervention’ and ‘efficacy rate’, potential sources of heterogeneity could not be determined from clinical perspective. It might be caused by methodological diversity of the identified RCTs such as study designs and risk of bias.

**Table 2:** Subgroup analysis and investigation of heterogeneity on SBP/DBP after intervention.

Outcome Title	Number of studies	Number of patients	Effect size WMD(95%CI)	Test for overall effect P-value	Heterogeneity; I^2^
SBP after intervention					
1.Type of intervention					
Acupuncture	7	510	-0.56 [-3.02, 1.89]	0.65	60%
Acupuncture plus western medicine	3	170	-9.04 [-20.11, 2.02]	0.11	94%
Acupuncture plus lifestyle	1	60	-10.53[-27.52, 6.46]	0.22	-
2.Number of sessions					
n≤20	5	310	-4.11 [-12.26, 4.05]	0.32	95%
n>20	6	430	-1.85 [-6.06, 2.36]	0.39	75%
3.Sample size					
n≤60	7	410	-5.42 [-11.91, 1.07]	0.10	95%
n>60	4	330	0.12 [-4.58, 4.81]	0.96	73%
4.Type of stimulation					
Electro	3	200	-1.56 [-4.59, 1.47]	0.31	23%
Manual	8	540	-4.05 [-10.36, 2.27]	0.21	95%
DBP after intervention					
1.Type of intervention					
Acupuncture	7	510	-1.01 [-2.26, 0.24]	0.11	23%
Acupuncture plus western medicine	3	170	-2.87 [-8.45, 2.72]	0.31	86%
Acupuncture plus lifestyle	1	60	-7.52 [-15.06, 0.02]	0.05	-
2.Number of sessions					
n≤20	5	310	-2.99 [-4.32, -1.66]	P<0.0001	85%
n>20	6	430	-1.80 [-2.97, -0.63]	0.003	42%
3.Sample size					
n≤60	7	410	-3.39 [-4.46, -2.33]	P<0.00001	75%
n>60	4	330	-0.06 [-1.61, 1.49]	0.94	0%
4.Type of stimulation					
Electro	3	200	-0.43 [-2.20, 1.34]	0.63	0%
Manual	8	540	-2.94 [-3.95, -1.93]	P<0.00001	78%

Abbreviation: SBP, systolic blood pressure; DBP, diastolic blood pressure; WMD, weighted mean difference; CI, confidence interval.

**Table 3:** Subgroup analysis and investigation of heterogeneity on SBP/DBP change magnitude.

Outcome Title	Number of studies	Number of patients	Effect size WMD(95%CI)	Test for overall effect P-value	Heterogeneity; I^2^
SBP change magnitude					
1.Type of intervention					
Acupuncture	2	216	0.30 [-0.27, 0.88]	0.30	0%
Acupuncture plus western medicine	2	170	-7.47 [-10.43, -4.51]	P<0.00001	0%
2.Number of sessions					
n≤20	2	218	-4.51 [-15.43, 6.42]	0.42	85%
n>20	2	168	-2.25 [-12.31, 7.81]	0.66	87%
3.Sample size					
n≤60	2	58	-3.55 [-17.55, 10.45]	0.62	85%
n>60	2	328	-3.19 [-10.32, 3.94]	0.38	95%
DBP change magnitude					
1.Type of intervention					
Acupuncture	2	216	-1.40 [-2.37, -0.44]	0.004	8%
Acupuncture plus western medicine	2	170	-4.22 [-6.26, -2.18]	P<0.0001	0%
2.Number of sessions					
n≤20	2	218	-2.68 [-6.55, 1.18]	0.17	52%
n>20	2	168	-1.97 [-6.65, 2.71]	0.41	73%
3.Sample size					
n≤60	2	58	-2.24 [-8.91, 4.43]	0.51	69%
n>60	2	328	-2.51 [-4.91, -0.12]	0.04	80%

Abbreviation: SBP, systolic blood pressure; DBP, diastolic blood pressure; WMD, weighted mean difference; CI, confidence interval.

**Table 4:** Subgroup analysis and investigation of heterogeneity on efficacy rate.

Outcome Title	Number of studies	Number of patients	Effect size WMD(95%CI)	Test for overall effect P-value	Heterogeneity; I^2^
1.Type of intervention					
Acupuncture	11	1023	1.14 [0.70, 1.85]	0.61	54%
Acupuncture plus western medicine	4	230	4.19 [1.65, 10.67]	0.003	0%
2.Number of sessions					
n≤20	7	498	0.96 [0.59 1.54]	0.85	18%
n>20	8	755	1.40 [0.98, 2.01]	0.04	69%
3.Sample size					
n≤60	10	590	2.13 [1.36, 3.34]	0.0009	28%
n>60	5	663	0.79 [0.53, 1.17]	0.24	63%
4.Type of stimulation					
Manual	2	140	1.32 [0.96, 1.80]	0.09	64%
Electro	13	1113	0.81 [0.39, 1.69]	0.58	0%

Abbreviation: WMD, weighted mean difference; CI, confidence interval.

### 3.5 Outcome and Effect Estimates

Based on various outcome measures (BP after intervention, BP change magnitude, Efficacy Rate) of the included RCTs, different pooled data of 22 RCTs were used in meta-analysis respectively. One trial [32] was excluded from meta-analysis because it reported BP value as medians and inter-quatile ranges. The effect estimates of acupuncture were shown in the forest plots (Figs 2–6).

All continuous data were presented as mean± SD. It is worth noting that, with respect to efficacy rate, it is often reported as a primary outcome by categorization of BP reduction in three levels (1.markedly effective, 2.effective, 3.inefficacious) in trials of hypertension in China. Efficacy rate means the percentage of the total number of participants who were categorized in the first two levels. And the criteria for categorization of the three levels are commonly defined as below in China [35]:

(1)Markedly effective: at the end of treatment, DBP decreased by 10 mmHg reaching the normal range, or DBP has not yet returned to normal but has been reduced ≥ 20 mmHg;(2)Effective: at the end of treatment, DBP decreased to less than 10 mmHg reaching the normal range, or DBP decreased by 10–19 mmHg but did not reach the normal range, or SBP reduction ≥ 30 mmHg);(3)Inefficacious: DBP reduction is not as significant as the first two levels at the end of treatment.

#### 3.5.1 BP after intervention

11 RCTs which reported BP (presented as mean ± SD) at the end of treatment as the primary outcome were pooled in the meta-analysis (as shown in Figs 2 and 3). 7 of them were divided into the subgroup of acupuncture versus western medicine. The result failed to establish a favorable effect of acupuncture on both SBP and DBP after intervention. (7 trials with 510 patients, SBP: MD = -0.56, 95%CI:-3.02 to 1.89, I^2^ = 60%; DBP: MD = -1.01, 95%CI:-2.26 to 0.24, I^2^ = 23%). 3 studies were combined as a subgroup analysis of acupuncture plus medication versus medication alone. Similarly, the result did not show a significant difference in favour of acupuncture, with significant heterogeneity. (3 trials with 170 patients, SBP:-9.04,-20.11 to 2.02, I^2^ = 94%; DBP:-2.87,-8.45 to 2.72, I^2^ = 86%). Compared with lifestyle modification, acupuncture was not superior on post-treatment SBP/ DBP as well. (1 trial with 60 patients, SBP: −10.53, −27.52 to 6.46; DBP: −7.52, −15.06 to 0.02)

#### 3.5.2 BP change magnitude

5 trial [19,21,32,33,34] reported SBP and DBP change magnitude(presented as mean ± SD) between baseline and post-intervention as principal outcomes and after excluding one trial[32]from meta-analysis because it reported BP value as medians and inter-quatile ranges,2 of them were pooled in the subgroup of acupuncture versus sham acupuncture. And it turned out that there were no significant differences between acupuncture and sham acupuncture (Fig 4: 2 trials with 216 patients, SBP: 0.30, -0.27 to 0.88, I^2^ = 0%) while acupuncture arms achieved more significant effect modification on DBP change magnitude than sham acupuncture, with low heterogeneity (Fig 5: 2 trials with 216 patients, DBP: -1.40, -2.37 to -0.44, I^2^ = 8%). The other 2 RCTs compared ‘acupuncture plus medication’ with ‘sham acupuncture plus medication’ and they were combined in this subgroup. The result yielded a beneficial effect of acupuncture combined with western medicine compared to sham acupuncture plus western medicine, with no heterogeneity. (Figs 4 and 5: 2 trials with 170 patients, SBP: -7.47, -10.43 to -4.51, I^2^ = 0%; DBP:-4.22, -6.26 to -2.18, I^2^ = 0%)

#### 3.5.3 Efficacy rate

14 RCTs reported efficacy rate as the main outcome and all data were pooled in the forest plot (Fig 6). Of these trials, 4 were pooled as a meta-analysis of acupuncture plus western medicine versus medication alone. And the outcome demonstrated that acupuncture as an adjunct to western medicine achieved more favourable effect than medication. (4 trials with 230 patients, OR = 4.19, 95%CI: 1.65 to 10.67, I^2^ = 0%).The other 10 RCTs were combined in the subgroup analysis of acupuncture versus western medicine. And the result failed to yield a beneficial effect of acupuncture. (10 trials with 963 patients, OR = 1.14, 95% CI: 0.70 to 1.85, I^2^ = 54%)

### 3.6 Risk of Bias Assessment

**Fig 7:**
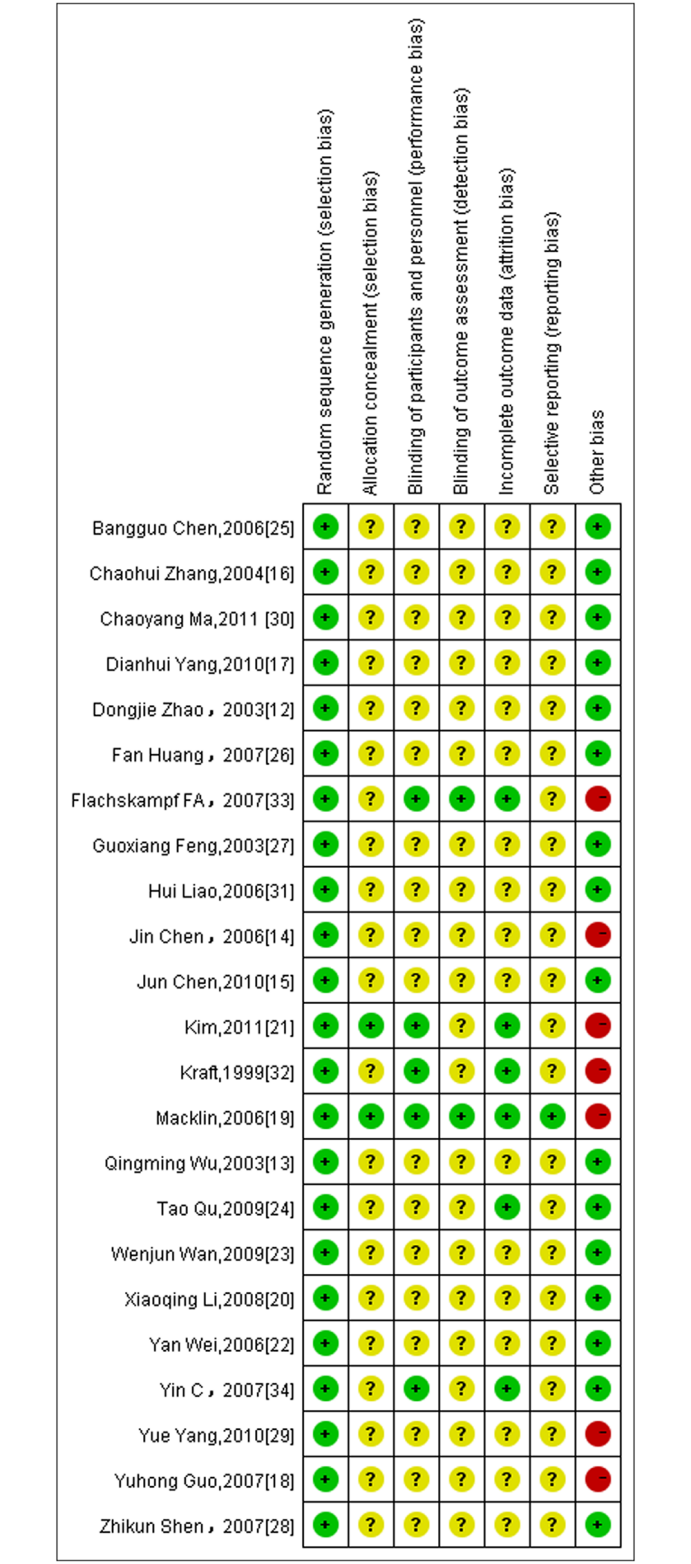
The risk of bias assessment for each included study.

**Fig 8:**
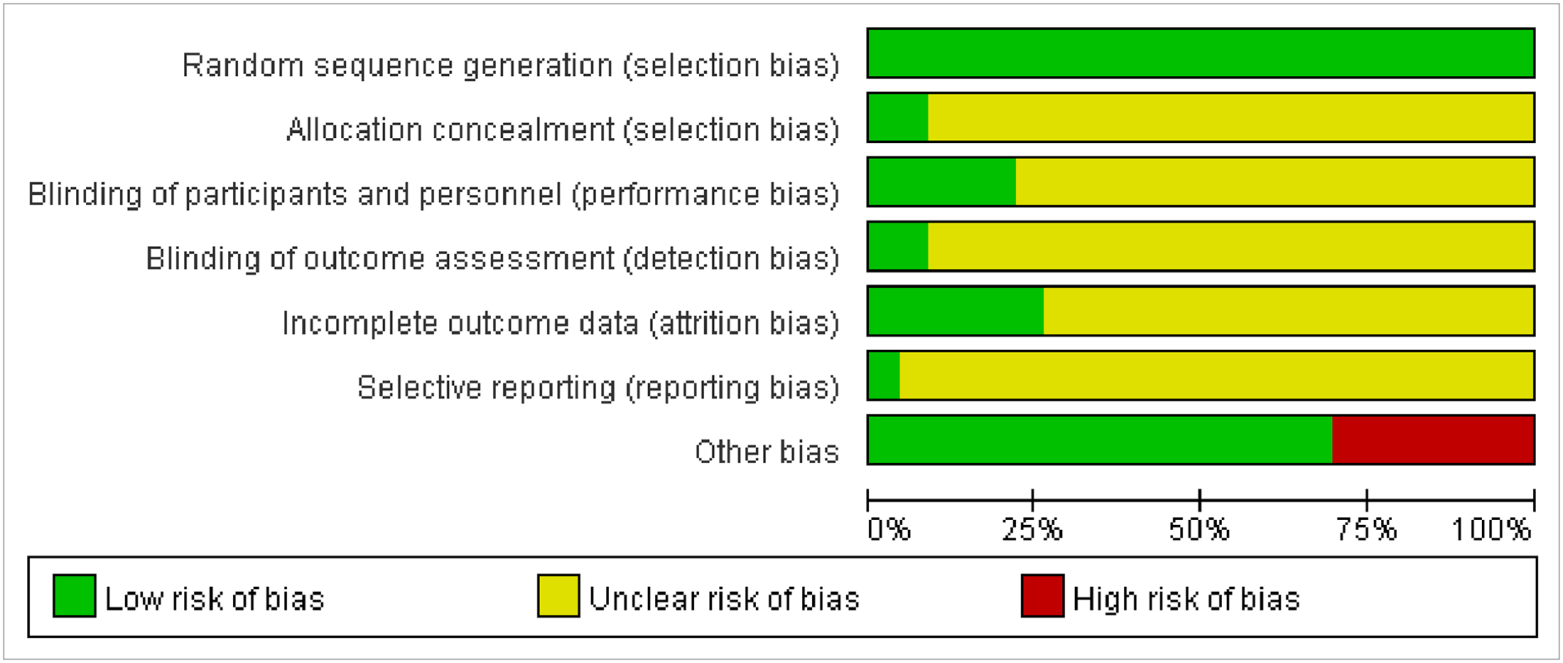
Risk of bias summary of included RCTs.

We summarized the risk of bias assessment for each included study in Figs 7 and 8, which was based on the Cochrane criterion [10]. The risk of bias of the included RCTs was generally low for random sequence generation and other sources of bias. The majority of the trials used table of random number to fulfill the random sequence generation, whereas 3 RCTs adopted block randomization[17,21,33] and 2 studies referred to the drawing method[29,31].

Details about allocation concealment were only mentioned in 2 RCTs [19,21], so the domain of allocation concealment was generally rated ‘unclear’ in most trials. With respect to blinding, only 3 trials had a description of double-blind [19, 21, 34] and single-blind was used in 2 trials [32, 33].

6 trials reported dropouts or withdraws [19,21,24,32,33,34], 4 of which provided the reasons[21,32–34] and 2 mentioned that outcomes were analyzed by intention-to-treat analysis[19,33]. Selective reporting was generally ‘unclear’ in the included RCTs due to the inaccessibility of the trail protocol.

Information on follow-up was addressed in 4 trials [19,21,28,33] and outcome in two trials showed BP gradually bounced to pre-treatment levels [19,33], while another study [28] didn’t find the occurrence of BP bounce.

### 3.7 Safety Evaluation

Most included RCTs had inadequate reporting on adverse events. Only 4 trials [19, 20, 33, 34] described them. One RCT reported spot-bleeding [34] and one [19] mentioned occurrence of study-related serious adverse events such as hypertensive urgencies and congestive heart failure during follow-up. And minor adverse event such as pain was recorded in one trial [33].

## Discussion

### Principal Findings

Our meta-analysis suggested that acupuncture plus western medicine had a more significant effect on both SBP and DBP change magnitude than sham acupuncture plus medication, which indicated that acupuncture has an add-on effect for western medicine. Similarly, regarding efficacy rate, outcome proved that acupuncture in combination with western medicine was superior to sole medication.

By contrast, no significant differences were found in acupuncture versus western medicine for lowing both SBP and DBP after intervention, which was also supported by the meta-analysis regarding efficacy rate. And compared with lifestyle modification, acupuncture had no beneficial effect on SBP/DBP after intervention as well. Similarly, there were no significant differences between acupuncture and sham acupuncture regarding SBP change magnitude while acupuncture arms achieved significant effect modification on DBP change magnitude compared with sham acupuncture.

On the basic of on the outcomes above, our review showed favorable evidence of acupuncture as an add-on therapy to western medicine for hypertension. And the evidence for acupuncture alone treating hypertension is insufficient. Additionally, the safety of acupuncture is still uncertain because of the poor description and we could not rule out the possibility of exclusion bias.

### Strengths and Limitation

Compared with previously published systematic reviews [4–7], pseudo-randomized trials simply claimed to be randomized with totally no description of random sequence generation were excluded in our review, thus only relatively rigidly designed RCTs were eligible. Besides, we also performed a meta-analysis regarding efficacy rate to evaluate effectiveness of acupuncture, which was absent in other 4 reviews [4–7]. Compared with the review of Zhao[9],we didn’t impose language restrictions during searching databases so 5 eligible RCTs[19,21,32–34] carried out in foreign countries were also included.

However, some limitations of our review should be addressed. Firstly, most included RCTs were published in Chinese journals so potential publication bias might exist due to different social cultural backgrounds. Chinese patients have a preference for acupuncture treatment compared to medical intervention and Asian countries are reported to be more likely to publish positive results in acupuncture researches [36]. Secondly, most eligible RCTs were classified as ‘unclear’ risk of bias of most domains. Especially, a majority of RCTs were in lack of the details of blinding which might lead to performance bias and detection bias, so the truth of these claimed RCTs would unavoidably be questionable. And substantial heterogeneity between studies was also detected in some subgroup analyses, with I^2^ values ranging from 0% to 94%. I^2^ has low statistical power with small numbers of studies and its confidence intervals could be large [11].Consequently, all current evidence might not be sufficiently robust against potential methodological flaws and significant heterogeneity. Thirdly, some included trials reported informal outcome measurement scales like efficacy rate. It means the percentage of the number of participants who are documented to achieve BP reduction markedly at the end of treatment. Regarding efficacy rate, the subgroup analysis showed that acupuncture plus medicine achieved more significant effect than medication alone. However, no significant differences were found in this subgroup regarding SBP and DBP after intervention. One plausible explanation for the conflicting results is that the currently definition of efficacy rate [35] is not very powerful and rational. It places more emphasis on the reduction of DBP to judge whether acupuncture is effective for treating hypertension. It may underestimate the importance of SBP reduction for hypertension. And this parameter is also relatively unclear and subjective because it could not reflect the extent of SBP/DBP reduction in precise measurement. So it is worth noting that, with respect to efficacy rate, the meta-analysis result should be interpreted in caution.

### Implications for Future Trials

Regarding the risk of bias assessment of the included RCTs using the Cochrane criteria [8], the majority of trials were evaluated as ‘unclear’ risk of bias in these domains: (1) allocation concealment(2) blinding of participants and investigators(3) blinding of outcome assessment(4) withdrawals/dropouts. Considering these defects of current trials, for future rigorously designed RCTs, these methodological domains should be assured. And in our review, risk of other bias in most trials published in Chinese journals was rated‘low’ but was assessed as ‘high’ in most Korea and American RCTs, which could be explained by the situation that in Chinese acupuncture trials, Deqi sensation are generally obtained because Chinese acupuncturists regard it as an important role in better efficacy according to theory of Traditional Chinese Medicine(TCM).

Regarding measurement of BP, trial evidence indicates that even small differences in SBP of 2–4mmHg are clinically important, thus accurate measurement is vital [37]. And 24-hour ambulatory blood pressure monitoring is vastly superior to the periodic non-automated blood pressure recordings using a cuff and sphygmomanometer. So we set an additional quality criterion of whether the RCTs involved 24-hour ambulatory monitoring and it turned out that only 3 trials [17, 21, 32] were qualified for this criterion.

As for acu-points selection, it is noteworthy that PC6 is well-known to have strong cardiovascular actions. Electric-acupuncture at the PC5–PC6 points had the potential to influence parasympathetic outflow and cardiovascular function in a rat model [38]. But we noticed that PC6 was not commonly used in the included RCTs. It turned out that only 4 trials used it [19,21,26,31]. Similarly finding was noted for ST9. On the basic of TCM theory, ST9 plays an important role in managing BP through ‘Qi’. Acupuncture at this point could stimulate the carotid sinus initiating the baroreflex. Thus, for future trials, some vital acu-points proposed in model studies should be taken into considerate account in acupuncture prescriptions during clinical practice.

With respect to manipulation method of acupuncture, as a TCM therapy, the effectiveness of acupuncture has close connection with acupuncturists' skills, competence and understanding of TCM theory [39]. After all, acupuncture is more than just sticking needles into specific points on the body. Considering it, some argued that acupuncture did lower blood pressure but the effect weaken or even lost when shoehorning it into a rigorously designed western-type clinical trial. Whereas, others criticized that the effect of acupuncture is not dependent on point locations or needling technique. The SHARP study bent over backwards to provide acupuncturists with diagnostic and treatment flexibility, yet no effect of acupuncture was found [19]. Patients were randomly assigned to each of 3 intervention groups using ‘individualized’ ‘standardized’ and ‘sham’ acupuncture. It turned out that all intervention groups experienced reductions in both SBP and DBP.

Besides, sham acupuncture was adopted as control in 5 of the RCTs [19,21,32–34]. However, sham acupuncture may not be appropriate as a placebo against which to evaluate the therapeutic effect of real acupuncture [40].Thus, seeking for an alternative sham control procedure may be a major goal in future acupuncture practice. Moreover, adequate serious consideration should be given in the future regarding other specific factors of acupuncture therapy, such as the treatment course, the frequency of sessions, since the dose of acupuncture is associated with the efficacy of acupuncture as well.

## Conclusion

In summary, our review provided evidence of efficacy of acupuncture as an adjunctive therapy to western medicine for treating hypertension, while the evidence for acupuncture alone lowing BP is insufficient. Additionally, the safety of acupuncture is still uncertain due to the inadequate reporting of it.

A major limitation was the generally unclear risk of bias of most methodological domains. Therefore, more rigorously designed and large-scale RCTs are necessary in the future, which should assure particularly adequate concealment of allocation and blinding of outcome assessors and adopt standardized measurements as the primary outcomes measured at long-term follow-up. Besides, with the presence of high heterogeneity of the included studies, a firm conclusion still could not be drawn on the efficacy of acupuncture for treating hypertension.

## Supporting Information

S1 Appendix PRISMA Checklist. (DOC)

S2 Appendix Search strategies. (DOC)
