# Supplementary material for: Is Acupuncture Effective for Hypertension? A Systematic Review and Meta-Analysis
Source: PLoS One. 2015 Jul 24;10(7):e0127019. doi: 10.1371/journal.pone.0127019 (PMC4514875; doi:10.1371/journal.pone.0127019)
Supplement: S2 Appendix — (DOC) [file pone.0127019.s002.doc]

**1. PUBMED (Search date: April 13, 2014)**

Search terms ：(("acupuncture"[MeSH Terms] OR "acupuncture"[All Fields] OR "acupuncture therapy"[MeSH Terms] OR ("acupuncture"[All Fields] AND "therapy"[All Fields]) OR "acupuncture therapy"[All Fields]) OR ("electroacupuncture"[MeSH Terms] OR "electroacupuncture"[All Fields])) AND (("hypertension"[MeSH Terms] OR "hypertension"[All Fields]) OR (essential[All Fields] AND ("hypertension"[MeSH Terms] OR "hypertension"[All Fields]))) AND (("random allocation"[MeSH Terms] OR ("random"[All Fields] AND "allocation"[All Fields]) OR "random allocation"[All Fields] OR "randomized"[All Fields]) OR ("clinical trials as topic"[MeSH Terms] OR ("clinical"[All Fields] AND "trials"[All Fields] AND "topic"[All Fields]) OR "clinical trials as topic"[All Fields] OR "trial"[All Fields]))

**2. EMBASE (Search date: April 13, 2014)**

Search terms ：'acupuncture'/exp OR acupuncture OR 'electroacupuncture'/exp OR electroacupuncture AND ('hypertension'/exp OR hypertension OR essential AND ('hypertension'/exp OR hypertension) OR 'blood'/exp OR blood) AND ('pressure'/exp OR pressure) AND (randomized OR trial)

**3. Cochrane Central Register of controlled trials (Search date: April 13, 2014)**

Search terms ：(acupuncture OR electroacupuncture) AND (hypertension OR essential hypertension) AND (randomized OR trial)

**4. International Clinical Trials Register Platform of WHO (Search date: April 13, 2014)**

Search terms：acupuncture AND hypertension

**5. Chinese Scientific Journal Database (Search date: April 13, 2014)**

Search terms:(针刺 OR 电针 OR 耳针 OR 针灸) AND (高血压 OR 原发性高血压) AND (随机 OR 对照 OR 研究)

**6. China National Knowledge Infrastructure (CNKI) (Search date: April 13, 2014)**

Search terms:(针刺 OR 电针 OR 耳针 OR 针灸) AND (高血压 OR 原发性高血压 ) AND (随机 OR 对照 OR 研究)

**7. Chinese Evidence-Based Medicine Database (Search date: April 13, 2014)**

Search terms: (针刺 OR 电针 OR 耳针 OR 针灸) AND (高血压 OR 原发性高血压)
